# Microbial Metagenomics Revealed the Diversity and Distribution Characteristics of Groundwater Microorganisms in the Middle and Lower Reaches of the Yangtze River Basin

**DOI:** 10.3390/microorganisms12081551

**Published:** 2024-07-29

**Authors:** Yue Wang, Ming-Yu Weng, Ji-Wen Zhong, Liang He, De-Jun Guo, Dong Luo, Jia-Yu Xue

**Affiliations:** 1 Lower Changjiang River Bureau of Hydrological and Water Resources Survey, Nanjing 210011, China; 15850573031@163.com (Y.W.); 13851830088@163.com (J.-W.Z.); hl1982liang@163.com (L.H.); 15715158867@163.com (D.-J.G.); 2 College of Horticulture, Bioinformatics Center, Academy for Advanced Interdisciplinary Studies, Nanjing Agricultural University, Nanjing 210095, China; 2023104086@stu.njau.edu.cn; 3 Fairy Lake Botanical Garden, Shenzhen & Chinese Academy of Sciences, Shenzhen 518004, China

**Keywords:** groundwater, microorganisms, metagenomics, diversity, antibiotic-resistant bacteria

## Abstract

Groundwater is one of the important freshwater resources on Earth and is closely related to human activities. As a good biological vector, a more diverse repertory of antibiotic resistance genes in the water environment would have a profound impact on human medical health. Therefore, this study conducted a metagenomic sequencing analysis of water samples from groundwater monitoring points in the middle and lower reaches of the Yangtze River to characterize microbial community composition and antibiotic resistance in the groundwater environment. Our results show that different microbial communities and community composition were the driving factors in the groundwater environment, and a diversity of antibiotic resistance genes in the groundwater environment was detected. The main source of antibiotic resistance gene host was determined by correlation tests and analyses. In this study, metagenomics was used for the first time to comprehensively analyze microbial communities in groundwater systems in the middle and lower reaches of the Yangtze River basin. The data obtained from this study serve as an invaluable resource and represent the basic metagenomic characteristics of groundwater microbial communities in the middle and lower reaches of the Yangtze River basin. These findings will be useful tools and provide a basis for future research on water microbial community and quality, greatly expanding the depth and breadth of our understanding of groundwater.

## 1. Introduction

Microorganisms, the most widespread and diverse group of organisms on Earth, play critical roles in biogeochemical cycles, food and biological resources, and environmental pollution remediation. Thus, they are often used as model organisms in studies of the Earth’s ecology and the environment [1]. Groundwater accounts for 30% of the Earth’s freshwater resources, second only to the polar ice cap in a frozen state [2]. Groundwater systems, as a key part of the Earth’s critical zone, are rich in microorganism diversity [3]. Human activities significantly impact groundwater ecosystems [4], rapidly altering the composition and abundance of microbial species. Therefore, studying the dynamic changes in groundwater microorganisms could serve as an important indicator of water ecological changes and help assess the specific impacts of human activities on groundwater quality.

In recent years, with the integration of disciplines and the development of biotechnology, metagenomics-based non-culture methods have allowed for unprecedented precision and comprehensiveness in quantifying and functionally characterizing the dynamic changes in environmental microbial systems [5,6,7]. Through metagenomic sequencing and statistical processing of bioinformatics technology, the genome reconstruction of previously unculturable microorganisms has been facilitated, enabling us to access vast microbial resources and their functional potentials from various environments. Currently, metagenomic research strategies have been widely applied in a diverse range of “environments,” from the miniature “environment” of the human gut [8] to the vast “environments” covering terrestrial and marine regions of the Earth [9]. However, due to the complex, variable, and invisible nature of groundwater environments, as well as the bottlenecks of in situ detection and long-term field monitoring, most studies have focused on surface water (including aquifers). These studies include microbial diversity in different regions and its response to environmental changes [4,10], while the microbial environment in groundwater has long been neglected, and metagenomic studies of groundwater are rare.

It has been found that microbial communities in groundwater interact with environmental factors, mediating geochemical element cycles (carbon, nitrogen, sulfur, etc.) in an orderly, interdependent manner [11,12,13]. Studies have shown that most (two-thirds) microbial carbon fixation pathways under natural conditions are found in groundwater systems [14,15]; microbial heterotrophic respiration can alter the composition of organic carbon in surface water–groundwater mixing zones [16,17]; ecological risk assessments can be conducted through understanding the diffusion of ARB (antibiotic-resistant bacteria) and ARGs (antibiotic resistance genes) in urban ponds [18]. Consequently, the interactive feedback between groundwater microorganisms and the environment has gradually become a research hotspot [19,20]. However, overall, research on groundwater systems is still relatively shallow and lacks a complete understanding of the feedback and evolution of microbial communities and their interactions with the environment.

In the microbial communities within urban aquatic habitats, both stochastic processes (species formation, birth, death, and immigration) and deterministic processes (interactions between the environment and microorganisms) play crucial roles in shaping the assembly of microbial communities [21,22]. In landscape ponds, stochastic processes largely contribute to the enrichment of prokaryotes [23]. However, the processes governing microbial community assembly in groundwater environments remain largely unknown. Similarly, the mechanisms underlying the accumulation and occurrence of antibiotic-resistant bacteria and their resistance genes in groundwater environments are still a black box. Deterministic biological and abiotic factors have significant effects on the aggregation of microbial communities and the persistence of resistance genes in aquatic environments [24]. Understanding these community-shaping processes, as well as the persistence of resistant bacteria and their genes, is of great significance for groundwater protection and monitoring.

Hence, this study leverages the existing national groundwater monitoring network to explore novel groundwater surveillance methods through metagenomic research. Taking the groundwater monitoring system in the middle and lower reaches of the Yangtze River as a case study, we aim to investigate the distribution, composition, and shaping mechanisms of groundwater microbial communities. This will pave the way for our subsequent biogeographical research expanding across the entire Yangtze River basin, experimenting with the dispersal patterns of groundwater microbes, contaminant dissemination, and microbial synthetic potential. By conducting prototype observations at the genomic level within groundwater environments, we will delve into the composition, dynamics, and interactions of groundwater microbial communities with environmental factors. This project will generate a vast amount of genetic and corresponding expression data, serving as a robust resource and tool for future research. It significantly enhances our understanding of groundwater systems and microbial community classification and conservation, providing a theoretical foundation for the green development of groundwater, as well as the scientific exploitation and safety of groundwater supplies.

## 2. Materials and Methods

### 2.1. Sample Collection

**Figure 1:**
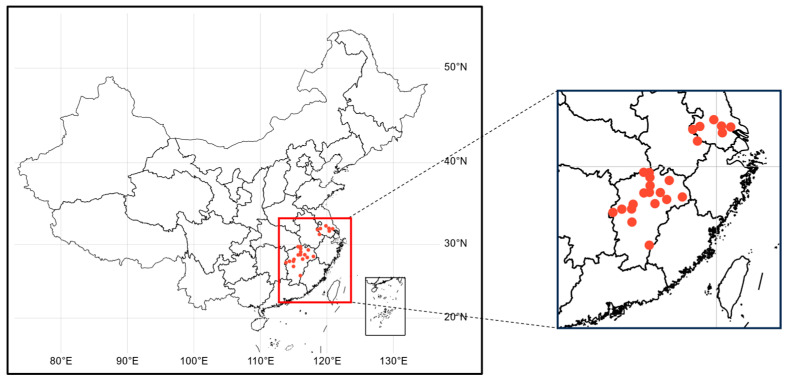
Geographic distribution of metagenomic samples from groundwater sampling points in Jiangxi and Jiangsu.

To elucidate the distribution and composition of microbial communities in the groundwater environment of the middle and lower reaches of the Yangtze River, in 2023, we analyzed historical water quality monitoring data and evaluation results spanning from 2018 to 2023 in cities such as Jiangxi and Jiangsu within this region. Following the classification of groundwater monitoring stations and water quality data based on the pollution status and distribution of the underlying aquifers, a total of 24 groundwater monitoring wells were selected as sampling sites to collect 24 water samples (Figure 1). Once the sampling points were finalized, the research relied on the groundwater quality monitoring efforts of the Hydrological Bureau to synchronously collect water quality and environmental samples from the selected sites. At local water environment monitoring centers, groundwater samples were collected through existing monitoring wells, with one sample collected from each location and named according to the respective region, resulting in a total of 24 water samples, including those from Jiangxi (n = 17) and Jiangsu (n = 7). During sampling, 800 mL of water was collected from each site using a sampler, and the sampling depths for each groundwater well are detailed in the Supplementary Materials (Table S1). The water samples were then collected in sterile plastic bottles and preserved in dry ice. Upon completion of water sample collection, insoluble impurities, aquatic animals, and plants were filtered out using filters. Subsequently, environmental DNA was extracted using the PowerWater DNA Isolation Kit(MO BIO, Carlsbad, CA, USA), followed by metagenomic sequencing. This comprehensive approach aims to deepen our understanding of the microbial communities present in the groundwater of the middle and lower reaches of the Yangtze River.

### 2.2. DNA Extraction, Metagenomic Sequencing, and Quality Analysis

Firstly, environmental genomic DNA was extracted from groundwater samples using the cetyltrimethylammonium bromide (CTAB) method. The filter membrane post-DNA extraction was cut into small pieces with scissors and placed into a 1.5 mL EP tube, where 200 μL of CTAB extraction buffer (consisting of 2% CTAB, 100 mmol/L Tris-HCl (pH = 8.0), 20 mmol/L EDTA, and 1.4 mol/L NaCl) was added and mixed thoroughly. Following this, the mixture was ground using a homogenizer and heated in a 70 °C water bath for 10 min to ensure complete DNA extraction. After DNA extraction via the separation method, an equal volume of chloroform/isoamyl alcohol was added to remove proteins. The extent of DNA degradation and potential contamination was monitored on 1% agarose gel. Following a DNA quality assessment, mechanical shearing and ultrasonication were employed to fragment the DNA, which was then subjected to purification, end repair, adapter ligation, and size selection via agarose gel electrophoresis to form a sequencing library. After library quality control, the qualified libraries were sequenced using the Illumina NovaSeq platform (China National GeneBank, Shenzhen, China). Initial data quality was inspected using Fastqc(version 0.12.0), and Trimmomatic (version 0.39) [25] with default parameters was utilized to filter and trim the reads, yielding 277.59 Gb of reads for further analysis (Table S2).

### 2.3. Bioinformatic Analysis

Using metagenomic test data as default parameters, species annotation was performed using Kraken2 [26] (version 1.2.1) and Bracken (version 2.6.2) Species in the samples were annotated to the species level by comparing clean reads with the marker gene set. After species annotation with Kraken2, Bracken was used to predict the actual relative abundance of species in the samples, thus obtaining species abundance of groundwater microorganisms at different taxonomic levels.

Genome assembly analysis was performed using MEGAHIT [27] (version: 1.2.9), resulting in contigs for the corresponding samples. ORF prediction of assembly results was conducted using Prokka [28] (version: v1.14.6). After gene prediction, redundancy was removed from the annotated genes using CD-HIT [29] (version: 4.6) with a similarity threshold of 95% and a coverage threshold of 90% to construct a non-redundant gene set. We used resistance Gene Identifier [30] (RGI, version 6.0.1) to annotate the protein sequences of non-redundant gene sets in the Antibiotic Resistance Database [31] (CARD, version 3.2.5). At the same time, ARG-OAPs [32] (version 3.0) was used to annotate and classify drug resistance genotypes. Finally, Plasflow [33] (version 1.1.0) was used to predict the location distribution of contig-carrying drug resistance genes.

### 2.4. Statistical Analysis

Based on species abundance, we used the Vegan R [34] package to calculate the Bray–Curtis distance matrix between sampling points and analyze beta diversity. Alpha diversity indices were calculated using USEARCH (version 10.0.24). NMDS analysis was performed to observe the similarity in microbial community composition among different sampling points. To understand the response of groundwater microbial communities to environmental factors and the assembly process of communities, we used the Neutral Community Model (NCM) to assess the potential impact of stochastic processes on microbial community assembly in the groundwater ecological environment [35]. The NCM emphasizes randomness and neutral processes in community structure, assuming that microbial individuals are ecologically identical with no competitive advantages. This model provides a theoretical basis for explaining microbial community formation and diversity in ecosystems lacking apparent selective pressures or environmental gradients, highlighting the importance of stochastic processes in community assembly. R^2^ in this model serves as an indicator of overall fit to the neutral model, with fit statistics calculated at a 95% confidence interval [36].

To explore the interactions within groundwater microbial communities and the correlation between microbial communities and antibiotic resistance genes, we constructed microbial ecological networks (MENs) using the igraph (version 1.3.5) and Vegan R [34] package with parameters set to r = 0.5, *p* = 0.05, and the method = Spearman. Network visualization was performed using Cytoscape (version 3.10.2). Redundancy analysis (RDA) was conducted using the Vegan R package [29] to perform multivariate multiple linear regression on response and explanatory variable matrices, followed by principal component analysis on the fitted value matrix. RDA was used to analyze the impact of environmental factors on microbial community abundance and structure in groundwater samples. Correlation heatmaps between microbial communities and groundwater physicochemical factors were generated using Spearman correlation calculations. The rest of the image was edited by ChiPlot (https://www.chiplot.online/, accessed on 5 June 2024).

## 3. Results

### 3.1. Distribution and Composition of Groundwater Microbial Communities

**Figure 2:**
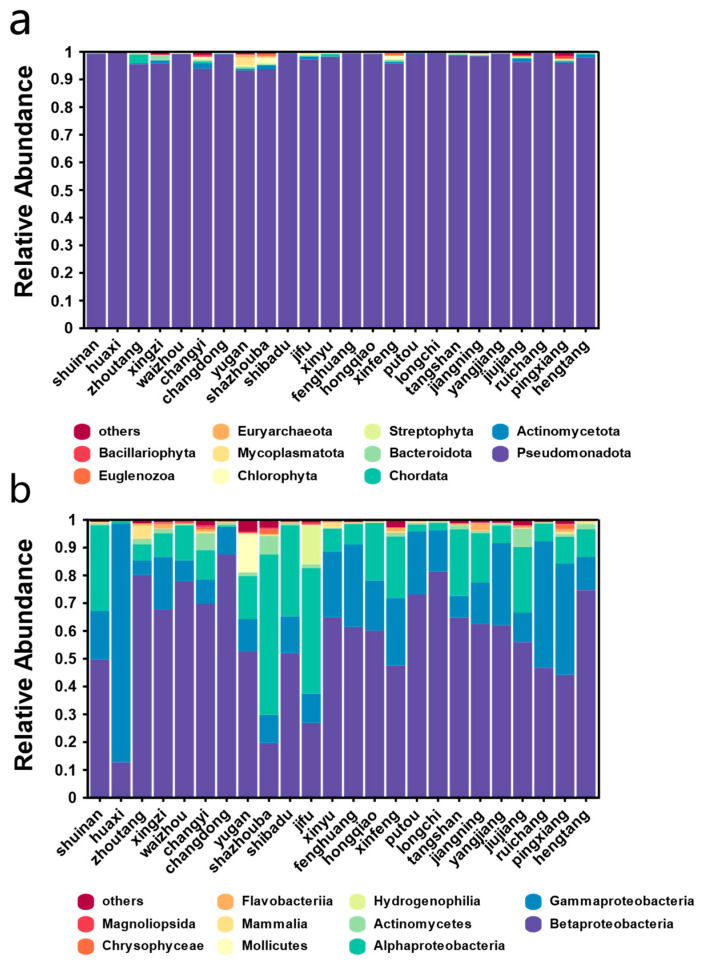
Composition of groundwater microbial communities. (**a**) Overview of microbial community structure at phylum level; (**b**) overview of microbial community structure at class level.

Groundwater is a critical source of water for agriculture, industry, and urban areas. Microorganisms within groundwater systems play significant roles in carbon neutrality and environmental remediation. The analysis revealed distinct differences in the composition of microbial communities. At the phylum level, *Pseudomonadota* predominated (Figure 2a), comprising over 97.86% of the total microbial counts, with an average relative abundance of 97.54% across all sites. Actinobacteria followed with a mere 0.41% abundance. The high enrichment of *Pseudomonadota* suggests significant pollution levels in groundwater sources. Further taxonomic classification at the class level identified *Alphaproteobacteria*, *Betaproteobacteria*, and *Gammaproteobacteria* as dominant (Figure 2b). Notably, Betaproteobacteria were predominant in most sites, *Gammaproteobacteria* were most abundant in Huaxi, *Alphaproteobacteria* dominated in Shazhouba and Jifu, and Mollicutes had the highest abundance in Yugan, accounting for 99.98% of Mollicutes in all samples.

**Figure 3:**
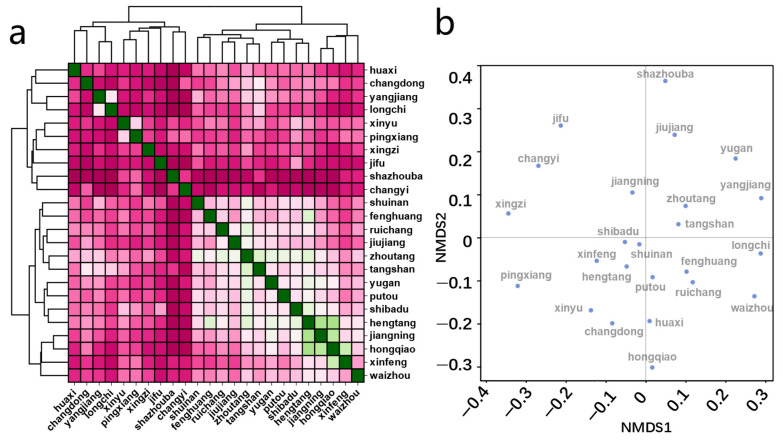
(**a**) Heatmap displaying cluster analysis based on distance matrix of species abundance, The difference in color represents the degree of similarity in species abundance between sample sites; (**b**) NMDS plot of groundwater microbial communities.

Next, cluster analysis was conducted based on the abundance of microbial community species (Figure 3a, Table S3) to explore whether there were differences in the composition of groundwater microorganisms in different regions. The figure shows that groundwater samples are divided into two categories, showing certain geographic heterogeneity. Additionally, we performed NMDS dimensionality reduction analysis to compare microbial diversity across regions. The NMDS plot (Figure 3b) demonstrates significant differences in microbial community composition under various groundwater environments, highlighting that distinct groundwater conditions shape diverse microbial communities. 

### 3.2. Microbial Community Response to Environmental Factors

**Figure 4:**
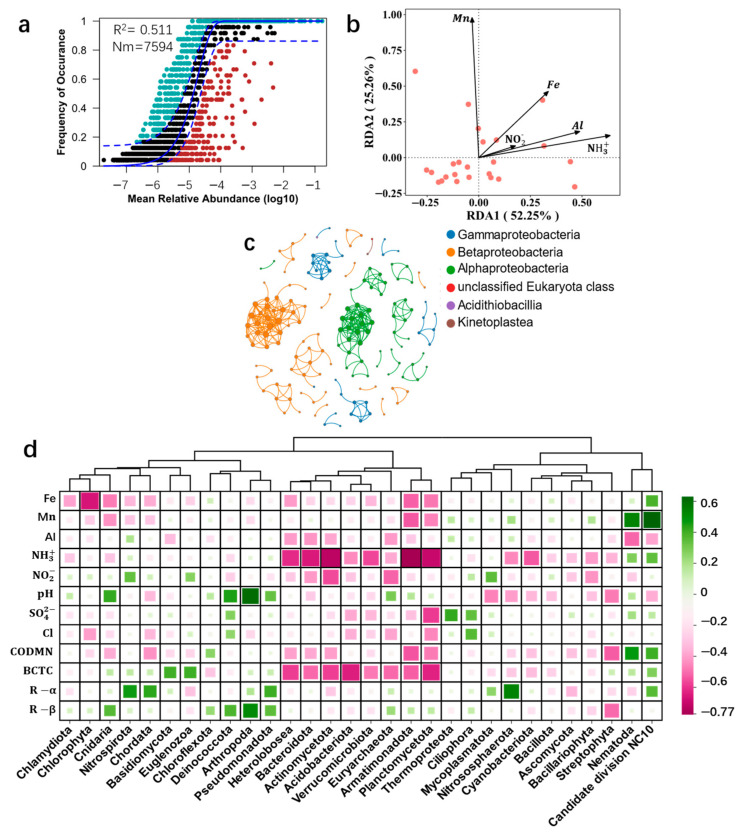
The response of groundwater microbial communities to environmental factors. (**a**) NCM (Neutral Community Model) fit of the groundwater microbial communities in Jiangxi and Jiangsu. The solid blue line represents the best fit with the NCM, and the dashed blue lines represent the 95% confidence interval predicted by the model. Microbial communities that occur more or less frequently than predicted by the NCM are shown in different colors. Nm represents the number of species in the groundwater microbial community, and BCTC (bacterial community total count) indicates the degree of model fit. (**b**) An ecological network diagram (MENs, microbial ecology networks) of groundwater microbial communities in Jiangxi and Jiangsu, constructed using Spearman correlation coefficients of relative abundance at the class level using kraken2 (version 2.1.2) (**c**) Redundancy analysis (RDA) of microbial communities at the phylum level and chemical factors in groundwater samples, with red dots representing Bray–Curtis distances between samples. The arrows indicate chemical factors influencing the composition of groundwater microbial communities. (**d**) A heatmap showing the correlation between physicochemical factors and microbial communities in groundwater.

To elucidate the factors driving the differences in groundwater microbial communities across regions, we employed the Neutral Community Model (NCM) to study community assembly. This model, based on neutral theory, emphasizes the randomness and neutrality in community structure, assuming that microorganisms are influenced solely by stochastic drift and migration. The R^2^ value of 0.511 (Figure 4a) indicates that random processes partially contribute to community aggregation, but environmental selection pressures also significantly shape microbial diversity. The selection pressure of microorganisms’ environment is uncertain. Specifically, the influence of factors such as metal ions, inorganic salts, temperature, and pH in the groundwater environment on the structural changes in microbial communities is unknown to us. Therefore, we will next explore the correlation between groundwater physicochemical factors and microbial communities.

To further uncover interaction patterns among microbial communities, we constructed a microbial ecological network (Figure 4b). The network revealed high modularity within microbial communities at the same taxonomic level, indicating strong and close interactions among groundwater microbial populations. These analyses suggest that unique microbial community structures form under different geographical environments. Using RDA analysis, we explored the driving factors of heavy metal elements and ammonium compounds on microbial communities, identifying potential relationships between environmental factors and microbial communities. The RDA analysis (Figure 4c) revealed significant correlations between groundwater microbial communities and elements such as Fe, Mn, Al, and ammonium compounds, with RDA1 and RDA2 explaining 52.25% and 25.2% of the bacterial community variations, respectively.

The unique structure of groundwater microbial communities and their interactions with environmental factors remain partially understood. To investigate how microbial communities respond to environmental changes, we performed Spearman correlation tests between the top 30 microbial phyla and 12 environmental factors (Figure 4d, Tables S4 and S5). The results show that primary water quality indicators like Al^3+^, NO_2_^−^, Cl^−^, and BCTC had weak correlations with microbial phyla. Fe^3+^ was negatively correlated with Chlorophyta, while Mn^2+^ showed a significant positive correlation with candidate division NC10. Ammonium (NH^3+^) was negatively correlated with phyla such as *Actinomycetota*, *Bacteroidota*, *Heterolobosea*, *Cyanobacteriota*, *Verrucomicrobiota*, *Planctomycetota,* and *Armatimonadota*. Total coliforms were significantly negatively correlated with phyla like *Actinomycetota*, *Bacteroidota*, *Euryarchaeota*, *Heterolobosea*, *Planctomycetota*, *Armatimonadota*, and *Acidobacterrota*. These findings suggest that environmental factors shape distinct groundwater microbial community structures.

### 3.3. Mechanisms of Antibiotic Resistance

**Figure 5:**
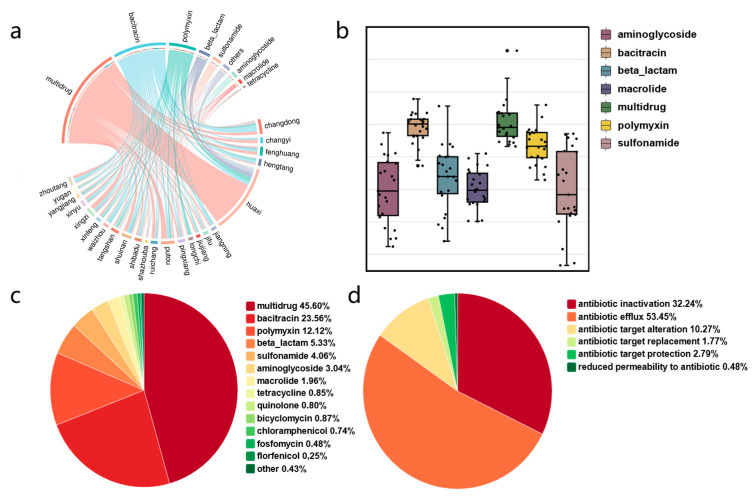
The occurrence of antibiotic resistance mechanisms. (**a**) The distribution of ARGs (antibiotic resistance genes) in groundwater samples from Jiangxi and Jiangsu. The lines and their thicknesses represent the types and quantities of ARGs detected at the sampling sites. (**b**) A box plot showing the number of different types of ARGs detected in groundwater samples; (**c**) species abundance of various resistance genes in groundwater samples; (**d**) the mechanisms of antibiotic resistance genes.

Groundwater serves as an essential source of water for various uses and a medium for environmental pollutant dissemination, with increasing pollution due to human activities, including the spread of antibiotic resistance genes (ARGs). To investigate the diversity and richness of antibiotic resistance mechanisms across different sites, we utilized the Comprehensive Antibiotic Resistance Database (CARD) [31] to predict ARGs by aligning non-redundant metagenomic gene sets. We identified 3371 ARGs in 24 different sites, spanning 892 subtypes and conferring resistance to 25 antibiotics (Figure 5a, Table S6). The predominant ARG types included multidrug (54.60%), bacitracin (23.56%), polymyxin (12.12%), beta-lactam (5.33%), sulfonamide (4.06%), and aminoglycoside (3.04%) (Figure 5b). These ARGs were classified into six mechanisms of action (Figure 5d): antibiotic efflux (53.45%), antibiotic inactivation (32.24%), antibiotic target alteration (10.27%), antibiotic target replacement (1.77%), antibiotic target protection (2.79%), and reduced permeability to antibiotics (0.48%). Multidrug resistance was the most representative ARG type, accounting for 45.60% of the total abundance (Figure 5c), with *bacA*, conferring bacitracin resistance, being the most common ARG. Notably, the abundance of multidrug ARGs was significantly higher in Huaxi than in other regions, suggesting a higher level of groundwater pollution in this area.

### 3.4. Mobility Potential of Antibiotic Resistance Genes in Groundwater

**Figure 6:**
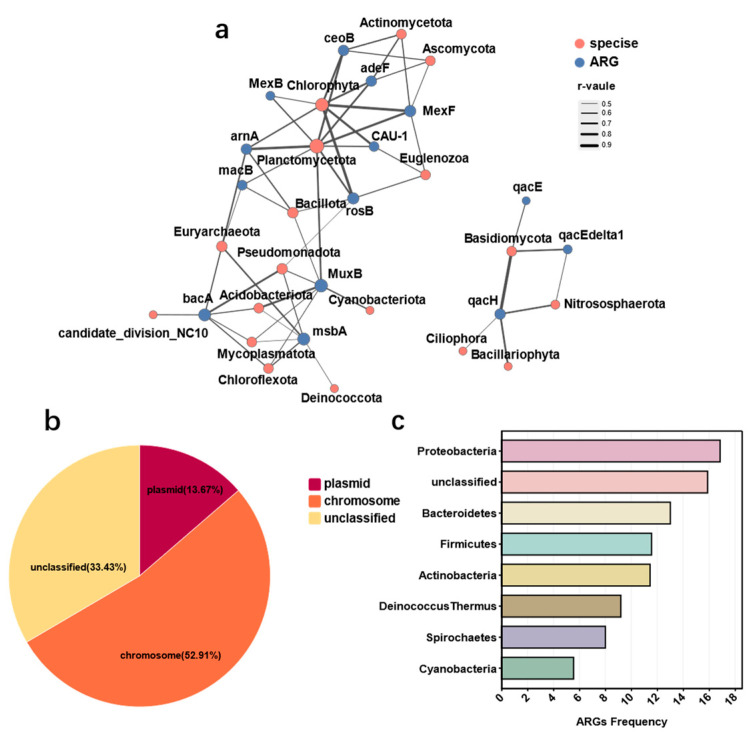
(**a**) Network analysis revealing co-occurrence of groundwater microbes and ARG subtypes. Node colors distinguish between microbial and ARG types, with line thickness and color indicating strength and nature of correlations. (**b**) Distribution and abundance of ARGs in plasmids and chromosomes within groundwater environment. (**c**) Correlation analysis between antibiotic resistance genes and their host microbial communities at phylum level in groundwater.

We explored the potential mobility of antibiotic resistance genes (ARGs) within groundwater environments by constructing a correlation network between microbial phyla and ARGs (Figure 6a, Table S7). The network revealed positive correlations between multidrug resistance genes *MuXB* and phyla such as *Chloroflexota* (r = 0.55), *Bacillota* (r = 0.55), *Cyanobacteriota* (r = 0.64), *Planctomycetota* (r = 0.70), and *Acidobacteriota* (r = 0.75), and of *msbA* with *Mycoplasmatota* (r = 0.51), *Acidobacteriota* (r = 0.51), *Deinococcota* (r = 0.52), *Chloroflexota* (r = 0.61), and *Euryarchaeota* (r = 0.68). Conversely, multidrug resistance genes *rosB* (r = 0.84), *MexF* (r = 0.83), *ceoB* (r = 0.74), and *MuXB* (r = 0.73) were negatively correlated with *Chlorophyta*. *Pseudomonadota*, the most abundant phylum in groundwater, was negatively correlated with the polymyxin resistance gene *rosB* (r = −0.50), the bacitracin resistance gene *bacA* (r = −0.57), and multidrug resistance genes *MuXB* (r = −0.58) and *msbA* (r = −0.73). The phylum *Planctomycetota* had the most associations with ARGs, including *macB*, *arnA*, *MuXB*, *rosB*, *CAU-1*, *MexF*, *MexB*, *adeF*, and *ceoB*. These results indicate that multidrug resistance genes have close relationships with groundwater microbial communities.

Groundwater, as a conducive medium for biological transmission, necessitates analyzing the mobility potential of microbial resistance genes. Microbial resistance can originate from intrinsic resistance (naturally present in bacterial genomes) or acquired resistance (horizontal gene transfer, HGT). Conjugation transfer, facilitated by mobile elements [37] like prophages, plasmids, and insertion sequences, is considered the most widespread HGT mechanism [38]. Most resistance genes can be disseminated through one or more horizontal transfer mechanisms, and nearly all accessory genetic elements identified in bacteria have the capability to capture resistance genes and facilitate their transfer [39]. To assess the mobility potential of ARGs in groundwater, we used Plasflow [33] to predict the genetic environment of resistance genes on chromosomes and plasmids (Figure 6b). Both chromosomes and plasmids were found to carry ARGs. Host correlation analysis at the phylum level (Figure 6c) indicated that ARG integration frequency was highest in *Proteobacteria* (61.01%).

## 4. Discussion

This study conducted a metagenomic sequencing analysis of groundwater samples collected from monitoring sites in the middle and lower reaches of the Yangtze River, with the aim of investigating the microbial community composition and antibiotic resistance gene (ARG) repertoire within the groundwater environment. Serving as an effective biological transmission medium, the aquatic environment exerts profound influences on human health and environmental safety. The research unveiled the presence of diverse microbial communities in groundwater, which is paramount to understanding the functionality and stability of groundwater ecosystems. Moreover, the abundant microbial communities in the aquatic environment provide a fertile ground for the exchange and transfer of resistance genes among antibiotic-resistant bacteria. Horizontal gene transfer (HGT) plays a pivotal role in enriching the ARG pool, contributing to its diverse landscape. Notably, the abundance of ARGs exhibits variability and complexity across different environmental media [40,41,42], underscoring the potential health risks posed by the diverse ARG repertoire in groundwater environments. This underscores the urgent need for intensified monitoring and management of groundwater quality to mitigate the threats posed by the dissemination of antibiotic resistance genes.

It is noteworthy that the environment has a profound impact on the dynamic assembly process of microbes [43,44]. Previous studies on the Yangtze River ecosystem have focused on the symbiotic relationships of bacterial communities in different habitats such as wetlands, soil, and sediments [45,46,47], while we focused on the microbial community composition in the aquatic environment, specifically investigating whether significant differences exist in groundwater microbial communities in different regions. Future research can further explore the interactions between groundwater microbial communities and environmental factors to better assess the health status of groundwater ecosystems. Additionally, this study comprehensively analyzed the microbial communities in the groundwater system of the middle and lower reaches of the Yangtze River using metagenomic approaches, providing new perspectives and avenues for research on groundwater ecosystems. Future studies can expand the sampling range to investigate and analyze groundwater across the entire Yangtze River basin, enhancing monitoring of the dynamic changes in groundwater microbial communities. At a large geographical scale, the topography of the Yangtze River basin plays a shaping role in the microbial communities in the water [48,49], and as the distance scale changes, microbial communities also undergo changes similar to those observed in other ecosystems such as the Tibetan Plateau [50,51,52]. Therefore, the next step will involve in-depth research on the correlation between groundwater microbial communities and geographical factors, which will contribute to a more comprehensive understanding of the functionality and stability of groundwater ecosystems, providing a scientific basis for the sustainable utilization and management of groundwater resources.

Our research has proven effective in identifying and analyzing the feedback interactions between known microorganisms in groundwater and environmental factors, revealing globally prevalent or underestimated microorganisms at the metagenomic level. However, a comprehensive analysis of the interplay between groundwater microbial communities and environmental factors necessitates further in-depth exploration along two dimensions: (a) horizontal studies (spatial dimension): dynamic monitoring of groundwater systems across the entire Yangtze River basin and other regions would be beneficial; (b) vertical studies (temporal dimension): high-frequency and long-term sequential studies will aid in monitoring fluctuations in microbial communities and establishing models correlated with relevant factors such as water quality, hydrology, urban population, pollution sources, and more. Such endeavors will enhance our understanding of the dynamic nature of these interactions and inform more effective strategies for groundwater quality management and conservation.

## 5. Conclusions

This study marks the first comprehensive analysis of microbial communities in the groundwater monitoring system of the middle and lower reaches of the Yangtze River utilizing metagenomic approaches. Our research showcases the diversity and shaping processes of groundwater microbial communities in this region, elucidating the occurrence and persistence characteristics of antibiotic-resistant bacteria in the groundwater environment. Within the microbial taxa, *Pseudomonadota* emerges as the dominant phylum, with varying compositions across different sites but strong interactions among microbial communities. In terms of the shaping effects of environmental physicochemical factors, a significant negative correlation is observed between metal ion Fe^3+^ and *Chlorophyta*, while Mn^2+^ positively correlates with *candidate division NC10*. Regarding the detection of antibiotic resistance genes (ARGs) in resistant bacteria, a total of 25 gene types conferring resistance to various antimicrobials were identified, with multidrug resistance (54.60%) being the primary category. The dominant mode of action for these resistance genes is antibiotic efflux (53.45%). Notably, *bacA*, conferring resistance to bacitracin, is the most prevalent ARG, while *MuxB*, belonging to the multidrug category, exhibits a close relationship with microbial communities. 

In summary, the groundwater environment harbors complex and diverse microbial communities, fostering intricate relationships between microbial communities and ARGs. The data generated from this preliminary study represent the fundamental metagenomic characteristics of groundwater microbial communities in the middle and lower reaches of the Yangtze River basin, which will guide future strategic groundwater resource reserves and facilitate systematic research on microbial community dynamics through temporal and spatial assessments across different regions. This work contributes to enhancing ecological risk monitoring of groundwater systems and offers ample opportunities for the advancement of groundwater science and the safe supply of groundwater resources.

## Data Availability

The raw genome sequencing data have been deposited in the China National Center for Bioinformation Database: GSA: CRA017218.

## Supplementary Materials

The following supporting information can be downloaded at https://www.mdpi.com/article/10.3390/microorganisms12081551/s1, Table S1. Groundwater detection location points and well depth; Table S2. Metagenomic sequencing data summary table for groundwater sampling points; Table S3. Distance matrix based on species abundance among groundwater sampling points; Table S4. The correlation matrix between groundwater microbial communities and environmental physicochemical factors; Table S5. Correlation matrix *p*-value between groundwater microbial communities and environmental physicochemical factors; Table S6. The annotation results of antibiotic resistance genes in groundwater sampling points; Table S7. The correlation between groundwater microbial communities and antibiotic resistance gene networks.
